# The Impact of Self-Reported Sleep on Caesarean Delivery in Women Undergoing Induction of Labour: A Prospective Study

**DOI:** 10.1038/s41598-017-12410-7

**Published:** 2017-09-26

**Authors:** Aimee Chuin Ai Teong, Annabella Xinhui Diong, Siti Zawiah Omar, Peng Chiong Tan

**Affiliations:** 1 Faculty of Medicine, University of Malaya, Lembah Pantai, Kuala Lumpur Malaysia; 2 Department of Obstetrics and Gynaecology, Faculty of Medicine, University of Malaya, Lembah Pantai, Kuala Lumpur Malaysia

## Abstract

216 women admitted for labour induction were recruited to evaluate sleep duration and other sleep measures on Caesarean delivery risk. The Pittsburgh Sleep Quality Index, Berlin (Obstructive Sleep Apnoea (OSA), Epworth Sleepiness Scale, International Restless Leg Syndrome, Insomnia Symptom Questionnaires were applied. Short sleep duration was defined as reported night sleep length in the previous month below the study population median of 6 hours. After binomial analysis, Caesarean delivery after labour induction is associated with short sleep duration (RR 1.8, 95% CI 1.1-2.9, P = 0.018), nulliparity, Bishop Score, prepregnant BMI and birth weight at P < 0.05. After adjustment for nulliparity, Bishop Score, prepregnant BMI and birth weight, short sleep duration remains independently predictive of Caesarean delivery AOR 2.4, 95% CI 1.1-5.0, P = 0.026. Women at high risk for OSA has a non-significant result on binomial analysis, RR 1.6, 95% CI 1.0-2.7, P = 0.073. In a sensitivity analysis which includes OSA in the multivariable logistic regression model, OSA’s predictive effect is attenuated AOR 1.2, 95% CI 0.4-3.2, P = 0.782 whilst short sleep duration remains significant AOR 2.3 95% CI 1.0-5.1, P = 0.039. Other evaluated sleep measures are not predictive of Caesarean delivery.

## Introduction

Lack of sleep has a major impact on general health: in a meta-analysis involving over a million subjects, short sleepers have a 12% greater risk of dying than those sleeping 7 to 8 hours per night^1^.

Across all months of pregnancy, poor sleep quality, insufficient night time sleep, daytime sleepiness, insomnia, sleep disordered breathing and restless legs syndrome are common^2^. In the third trimester as compared to early pregnancy, short sleep duration increases from 26.2% to 39.9% and poor overall sleep quality increases from 39.0% to 53.5%^3^.

Short sleep duration during pregnancy is associated with adverse outcome. Nulliparous women who slept less than 6 hours at night were 4.5 times more likely to have Caesarean delivery and to have longer labours^4^. Women who slept less than 7 hours at night have increased risk of developing gestational diabetes^5^. Sleep duration 6 hours or less at night in early pregnancy is associated with increased mean blood pressure in the third trimester^6^. Sleep duration of 5 hours or less per night is a risk factor for preterm birth^7^.

Obstructive sleep apnoea in pregnant women is also associated with more frequent preeclampsia, preterm birth, Caesarean delivery and neonatal intensive care unit admission^8^.

Induction of labour is an increasingly common obstetric intervention. In the United States, the rate of induction of labour more than doubled from 1990 through 2010, from 9.6% to 23.8%^9^. A 2014 meta-analysis of 31 trials finds that a policy of induction at 37-42 weeks of gestation is associated with a significant reduction in the risk of Caesarean section compared with expectant management^10^. This finding may precipitate a further increase in the rate of labour induction.

We hypothesize that preceding short sleep duration will negatively impact the response to labour induction resulting in a higher Caesarean delivery rate. We sought to evaluate the association of short sleep duration and other sleep measures to Caesarean delivery in women undergoing labour induction.

## Methods

This study was conducted in full compliance with the principles proclaimed in the Declaration of Helsinki on human research. Consent was informed and collected in the written format from all participants. Recruitment was from 3^rd^ November 2014 to 29^th^ April 2015.

### Sample size calculation

We opted to dichotomize our participants to short versus good sleep duration categories based on the 50th centile (median) cut-off for reported night sleep within our study population. As data on the effect of short sleep duration on Caesarean delivery following induction of labour is not available, we use as guidance an earlier paper investigating sleep duration in pregnancy and labour outcomes^4^; women in that study within the approximate upper half of sleep duration compared to women in the lower half has a Caesarean delivery rate of 35.1% (20/57) v. 10.8% (8/74). Applying the Chi Square test, alpha = 0.05, power = 0.8, and P_0_ = 10.8%, P_1_ = 35.1% and a case to control ratio of 1 to 1, a total of 92 participants are required for a powered study based on these parameters^11^. As we planned to perform multivariable logistic regression analysis with likely multiple predictor variables incorporated in the model, we aim to have 50 Caesarean deliveries in our study population (sufficient for analysis of 5 predictor variables based on the 10 events per predictor variable rule for multivariable logistic regression analysis^12^). A previous report from our hospital showed a 26.1% (69/264) Caesarean section rate after labour induction in a mixed population of nulliparas and multiparas^13^. A sample size of 192 (50/0.261) is calculated to attain 50 Caesarean deliveries. Building in a 10% margin for drop-outs, we aimed to recruit at least 213 (192/0.9) participants.

### Questionnaires

The following sleep related questionnaires were used in an interview around the start of induction: Pittsburgh Sleep Quality Index (PSQI), the Epworth Sleepiness Scale (ESS), Berlin Questionnaire for Sleep Apnoea, the International Restless Legs Syndrome (IRLS) Rating Scale, and the Insomnia Symptom Questionnaire (ISQ). These tools are used in combination in recent studies to assess the global impact of sleep on pregnancy outcome^2,3,14^.

The PSQI (19-item questionnaire) is validated for use in pregnancy^15^ and assessed sleep quality over the last month. A global score >5 indicates poor sleep quality and it has been shown to have a diagnostic sensitivity of 89.6% and a specificity of 86.5% in distinguishing between good and poor sleepers^16^. Sleep duration was obtained from a stem answer within the PSQI questionnaire; the hours of sleep at night was recorded in whole numbers and referred to sleep duration in the last month.

The ESS is a widely used measure of recent daytime sleepiness, with scores ranging from 0 to 24. Excessive daytime sleepiness is defined as a total score of ≥10^17^. It is validated in first trimester pregnancy^18^, and used in several last trimester sleep studies^2,3^.

The Berlin Questionnaire is scored in 3 categories; when a participant has at least 2 categories with a positive score, the case categorization is high risk for obstructive sleep apnoea (OSA)^19^. Berlin sleep questionnaire is predictive in later pregnancy for obstructive sleep apnoea^20^.

IRLS Rating Scale is a validated rating scale for Restless Leg Syndrome in the past 1 week, comprising of 10 questions which produces an overall score could range from 0 to 40^21^. We opted to dichotomize our participants to absence (score 0) or presence of Restless Leg Syndrome (score 1 to 40).

The ISQ is a 13-item self-report instrument designed to identify insomnia in the past month and is validated in pregnant women. Respondents are classified into insomnia disorder and no insomnia disorder^22^.

### Recruitment

Women were recruited by co-authors (AT and AD) based on investigator availability basis on their admission for planned labour induction. Other inclusion criteria are reassuring fetal status on pre-induction cardiotocogram, 37 to 42 weeks’ gestation, age 18 to 45 years, singleton fetus, intact membranes and cephalic presentation. The exclusion criteria are previous caesarean section, intrauterine fetal death or known gross fetal anomaly, known pre-pregnancy sleep or psychiatric disorders.

Patients’ clinical notes were scrutinised for eligibility. A patient information sheet was supplied to all women who were approached for recruitment. The recruiter answered any oral queries. Written consent was obtained from all participants. Demographic, clinical and questionnaire data were collected.

Labour induction for study participants follows our standard institution protocol. Briefly, patients are typically admitted at about 8 o’clock on the morning of induction. Cardiotocography is performed and induction of labour is only started if the cardiotocograph is reassuring. Method of induction of labour is decided as guided by cervical favourability. Dinoprostone (3 mg) pessary is typically used for cervical ripening and if the cervix is favourable, amniotomy is performed followed by titrated oxytocin infusion to induce labour. The details of the induction protocol is described in a published study from our centre^13^.

Responses to study questionnaires were not revealed to care providers. The investigators were not involved in the care of the participants. Post-induction outcomes were retrieved from medical notes after delivery.

### Analysis

Data were entered into statistical software package SPSS version 22.0 (SPSS Inc., Chicago, IL, USA). Analysis was performed after dichotomisation into Caesarean delivery vs. vaginal delivery after labour induction. A binomial analysis was first performed to evaluate predictors of Caesarean delivery from relevant demographics and sleep questionnaires’ findings. We planned to include all predictors with p < 0.05 on binomial analysis in the model for our primary multivariable logistic regression analysis. We expressed the data as mean and standard deviation for normally distributed continuous measures, median and interquartile range for ordinal or non-normally distributed continuous variables and frequencies and proportions for categorical measures. Chi square test was used for analysing differences in proportions for categorical variables. Student t test and Mann Whitney U test was used to analyse means for normally distributed and ordinal or non-normally distributed continuous variables respectively. P < 0.05 is taken as the level of statistical significance.

### Ethics approval

Ethics oversight was provided by the Medical Ethics Committee of University of Malaya Medical Centre who granted approval on 11 September 2014 (MECID No. 20147-371).

### Data Availability

Data may be made available on request subject to approval by the Ethics Committee of the University Malaya Medical Centre on participants’ confidentiality.

## Results

**Figure 1:**
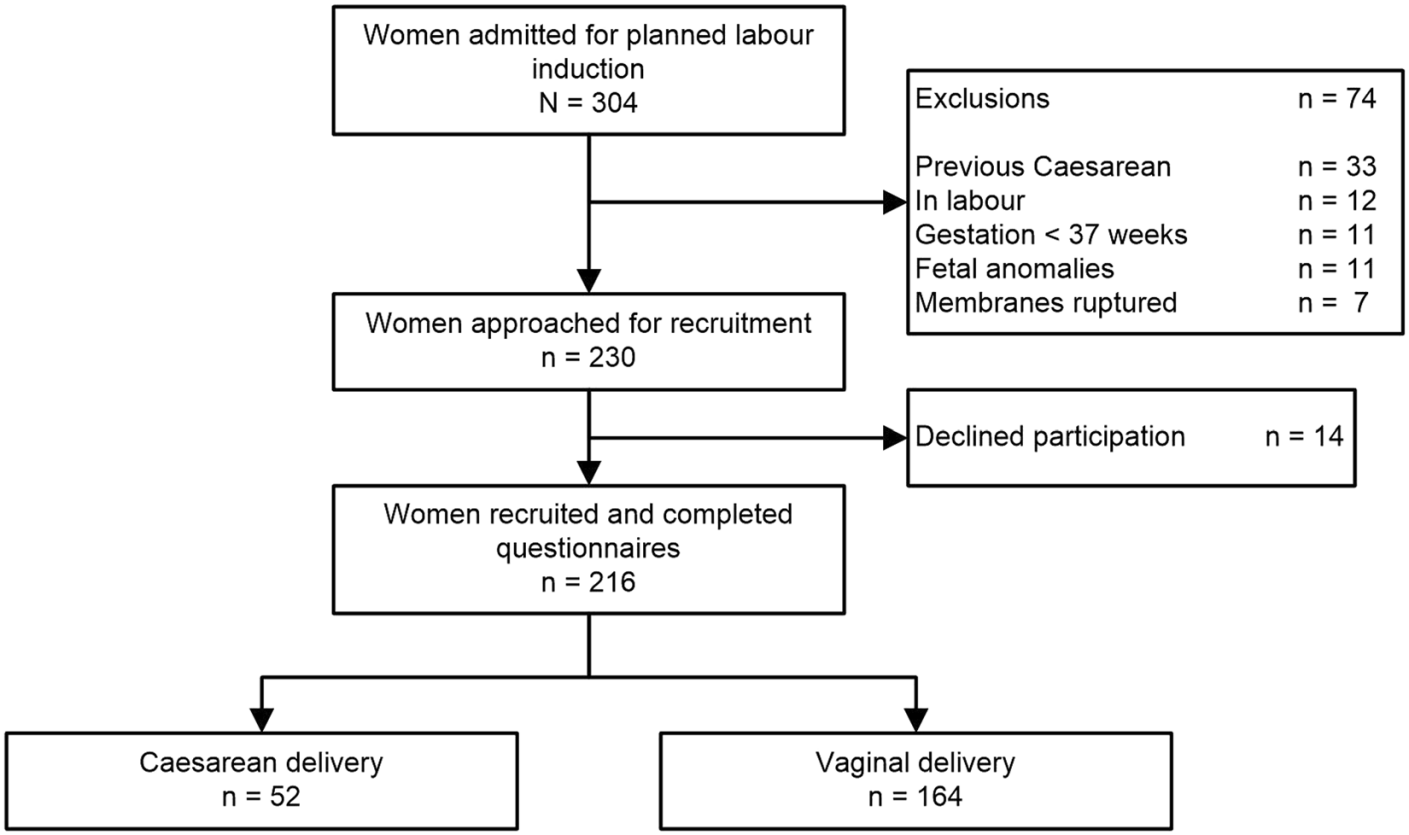
Recruitment Flow chart for a Prospective Study in Women Undergoing Labour Induction on Impact of Sleep Duration and Quality on Caesarean delivery.

The recruitment and flow through the study is depicted in Fig. 1. 216 respondents were recruited. We stopped recruitment on achieving our target sample size.

Table 1 shows the baseline characteristics of the study population including the findings of the sleep questionnaires. The median (approximate 50^th^ centile) reported night sleep duration for our study population was 6 hours. Therefore based on a priori 50^th^ centile cut-off, we classify short sleep duration as <6 hours and good sleep duration as ≥6 hours; 47.2% reported less than 6 hours of sleep a night during the previous month.

**Table 1:** Characteristics of study population.

Characteristic	Participants (N = 216)
Age(years, mean ± SD)	31.2 ± 4.5
Nulliparous	118 (54.6)
**Ethnicity**	
Malay	129 (59.7)
Chinese	29 (13.4)
Indian	43 (19.9)
Others	15 (6.9)
Prepregnancy BMI (kg/m^2^, mean ± SD)^*^	25.1 ± 5.7
Gestational Weight Gain (kg/m2, mean ± SD)^*^	13.2 ± 5.7
Gestational age (weeks, mean ± SD)	39.3 ± 1.3
**Indication of labour induction**	
Diabetes	98 (45.4)
Prolonged pregnancy	49 (22.7)
Hypertension	25 (11.6)
Non-reassuring fetal status^†^	37 (17.1)
Others	7 (3.2)
Bishop score (median [IQR])	2 [1–3]
**Method of labour induction**	
Vaginal dinoprostone^‡^	201 (92.6)
Amniotomy	15 (6.9)
**Reported Night Sleep**	
Duration (hours, median [IQR])	6 [5–7]
**Median cut-off**	
Short Sleep Duration <6 hours	102 (47.2)
Good Sleep Duration ≥6 hours	114 (52.8)
**Quartile cut-offs**	
< 4 hours	46 (21.3)
5 hours	56 (25.9)
6 hours	53 (24.5)
≥7 hours	61 (28.2)
**Pittsburgh Sleep Quality Index**	
Poor sleep quality	150 (69.4)
Good sleep quality	66 (30.6)
**Berlin Risk of Sleep Apnea** ^**§**^	
High risk	41 (19.0)
Low risk	172 (79.6)
**Epworth Sleepiness Scale**	
Daytime sleepiness	33 (15.3)
Normal	183 (84.7)
**Insomnia Symptom Questionnaire**	
Insomnia	40 (18.5)
None	176 (81.5)
**Restless Leg Syndrome**	
Present	120 (55.6)
Absent	96 (44.4)
Birth Weight (kg, mean ± SD)	3.0 ± 0.5

Values are stated as n (%), median [Interquartile Range, IQR] or mean ± standard deviation. ^*^n = 205 (11 women were unable to recall prepregnancy weight/gestational weight gain/height). ^†^Includes oligohydramnios, suspected IUGR, reduced fetal movement and suboptimal umbilical artery on Doppler examination. ^‡^Includes a single case of ripening with Foley catheter. ^§^n = 213 (Unable to classify 3 women sleep apnea risks due to missing BMI data).

Table 2 depicts the predictor characteristics dichotomized to all-cause Caesarean delivery compared with vaginal delivery (inclusive of spontaneous and instrumental) after induction of labour. On binomial analysis, Caesarean delivery after labour induction is significantly associated (P < 0.05) with nulliparity, pre-pregnancy BMI, Bishop Score, short sleep duration (<6 hours) and birth weight. Short sleep duration has a relative risk (RR) of 1.8, 95% Confidence Interval (CI) 1.1 – 2.9; P = 0.018 for Caesarean delivery. Berlin OSA stratification to high risk for OSA is a non-significant result RR 1.6, 95% CI 1.0-2.7; P = 0.073 as predictor of Caesarean delivery. Poor sleep quality (assessed by PSQI), day time sleepiness (assessed by ESS), presence of symptoms of restless leg syndrome (assessed by IRLS Rating Scale) and insomnia (assessed by ISQ) were not predictive of Caesarean delivery. Following multivariable logistic regression incorporating the five aforementioned variables with binomial analysis P < 0.05, short sleep duration adjusted odds ratio (AOR) 2.4, 95% CI 1.1-5.0, P = 0.026 remains predictive of Caesarean delivery.

**Table 2:** Participants’ Characteristics Dichotomized into Caesarean Delivery and Vaginal Delivery Groups.

Variable	Caesarean Delivery (n = 52)	Vaginal Delivery (n = 164)	P Value	RR (95% CI)	Multivariable Logistic Regression Analysis	
					AOR (95%CI)	P value
Age (years, mean ± SD)	30.8 ± 3.9	31.4 ± 4.7	0.438			
Nulliparity	43 (82.7)	75 (45.7)	<0.001	4.0 (2.0–7.7)	11.9 (4.2–33.5)	<0.001
**Ethnicity**						
Malay	28 (53.8)	101 (61.6)	0.050			
Chinese	4 (7.7)	25 (15.2)				
Indian	17 (32.7)	26 (15.9)				
Others	3 (5.8)	12 (7.3)				
Prepregnancy BMI (kg/m2, mean ± SD)^*^	27.1 ± 5.8	24.6 ± 5.6	0.007		1.1 (1.0–1.2)	0.002
Gestational Weight Gain (kg/m2, mean ± SD)^*^	13.4 ± 5.4	13.1 ± 5.8	0.772			
Gestational age (weeks, mean ± SD)	39.4 ± 1.4	39.2 ± 1.3	0.413			
**Indications for Induction of Labour**						
Diabetes mellitus	26 (50.0)	72 (43.9)	0.113			
Prolonged pregnancy	15 (28.8)	34 (20.7)				
Hypertension	7 (13.5)	18 (11.0)				
Non-reassuring fetal status^†^	4 (7.7)	33 (20.1)				
Others	0 (0.0)	7 (4.3)				
Bishop score (median [IQR])	1 [1-2]	2 [1-3]	0.012		1.0 (0.7–1.3)	0.812
**Method of labour induction**						
Vaginal dinoprostone^‡^	49 (94.2)	152 (92.7)	0.702	1.2 (0.4–3.5)		
Amniotomy	3 (5.8)	12 (7.3)				
**Reported Night Sleep**						
Duration (hours, median [IQR])	5 [4–6]	6 [5–7]	0.093			
**Median cut-off**						
Short Sleep Duration < 6 hours	32 (61.5)	70 (42.7)	0.018	1.8 (1.1–2.9)	2.4 (1.1–5.0)	0.026
Good Sleep Duration ≥ 6 hours	20 (38.5)	94 (57.3)			1	
**Quartile cut-offs**						
<4 hours	14 (26.9)	32 (19.5)	0.062^§^			
5 hours	18 (34.6)	38 (23.2)				
6 hours	8 (15.4)	45 (27.4)				
≥7 hours	12 (23.1)	49 (29.9)				
**Pittsburgh Sleep Quality Index**						
Poor Sleep Quality	35 (67.3)	115 (70.1)	0.701	0.9 (0.5–1.5)		
Good Sleep Quality	17 (32.7)	49 (29.9)				
**Berlin Risk of Sleep Apnea** ^||^						
High Risk	14 (28.0)	27 (16.6)	0.073	1.6 (1.0–2.7)		
Low Risk	36 (72.0)	136 (83.4)				
**Epworth Sleepiness Scale**						
Daytime Sleepiness	10 (19.2)	23 (14.0)	0.363	1.3 (0.7–2.4)		
Normal	42 (80.8)	141 (86.0)				
**Restless Leg Syndrome**						
Present	29 (55.8)	91 (55.5)	0.972	1.0 (0.6–1.6)		
Absent	23 (44.2)	73 (44.5)				
**Insomnia Symptom Questionnaire**						
Insomnia Disorder	11 (21.2)	29 (17.7)	0.574	1.2 (0.7–2.1)		
None	41 (78.8)	135 (82.3)				
Birth Weight (100 grams, mean ± SD)	31.5 ± 4.5	29.9 ± 4.6	0.025		1.1 (1.0–1.2)	0.012

Values are stated as n (%), median [Interquartile Range, IQR] or mean ± standard deviation. Analysis was by t-test for continuous variables, Mann-Whitney U Test for ordinal data, Chi-square test for categorical data, and chi-square test for trend for sequential categorical data. Multivariable logistic regression analysis on 205 respondents were used to identify independent predictors for caesarean delivery using all variables with a crude p < 0.05. Adjusted odd ratio is shown for all variables used in the model. *n = 205 (11 women were unable to recall prepregnancy weight/gestational weight gain/height). ^†^Includes oligohydramnios, suspected IUGR, reduced fetal movement and suboptimal umbilical artery on Doppler examination. ^‡^Includes a single case of ripening with the Foley catheter. §p value obtained from Chi Square for trend. ^||^n = 213 (Unable to classify 3 women into high or low risk for sleep apnea due to missing BMI data).

Post hoc, a sensitivity analysis was performed incorporating predictor characteristics with P < 0.1 (including Berlin OSA and ethnicity) on binomial analysis to check whether the finding of short sleep duration as a predictor for Caesarean delivery after labour induction is robust after adjustment for a larger number of potential confounders. Short sleep duration’s AOR 2.3, 95% CI 1.0-5.1, P = 0.039 for Caesarean delivery is not materially changed in this model. The predictive value of obstructive sleep apnoea is severely attenuated after adjustment for short sleep duration and other potential confounders AOR 1.2 95% CI 0.4-3.2; P = 0.782 (Supplementary Table 1).

Caesarean delivery rates after labour induction were 43/118 (36.4%) and 9/98 (9.2%) in our nulliparous and multiparous participants respectively. We performed an exploratory analysis based on nulliparous women at high risk for Caesarean delivery to evaluate the effect of sleep measures (Supplementary Table 2). Despite an almost halving of the sample size on restricting analysis to only nulliparas which might have reduced statistical power, on binomial analysis, the association of short sleep duration to Caesarean delivery was RR 1.9, 95% CI 1.2-3.2, P = 0.008 and high risk OSA RR 1.8, 95% CI 1.1-2.9, P = 0.036 were significant at the P < 0.05 level. The median sleep duration was also significantly shorter (5 [IQR 4-5] vs. 6 [IQR 5-7], P = 0.018) and the Chi Square for trend (after categorization into sleep duration quartiles) shows a significant inverse trend (P = 0.014) in nulliparas who had Caesarean delivery compared to vaginal delivery after labour induction. Incorporating short sleep duration, high risk OSA, prepregnant BMI, birth weight, Bishop Score and ethnicity into the multivariable logistic regression analysis model, short sleep duration remains independently predictive for Caesarean delivery AOR 2.8, 95% CI 1.1-7.1, P = 0.031 whilst the predictive value of being at high risk OSA is attenuated AOR 1.2, 95% CI 0.4-4.3, P = 0.75. The impact of short sleep duration in nulliparas for predicting Caesarean delivery after labour induction appears marginally stronger than when the entire mixed nulliparous and multiparous study population was considered AOR 2.8, 95% CI 1.1-7.1 v AOR 2.3, 95% CI 1.0-5.1.

Table 3 shows the correlation of sleep duration with selected other characteristics and labour outcomes as post hoc exploration. With short sleep duration, the induction to delivery interval (after excluding Caesarean delivery indicated by non-reassuring fetal status) is longer by an average 4 hours; (mean ± standard deviation) 23.9 hours ± 13.3 vs 19.9 hours ± 12.6, p = 0.036. On binomial analysis, short sleep duration is inversely associated with low birth weight ( < 2.5 kg) RR 0.4, 95% CI 0.2-1.0, P = 0.048, higher gestational weight gain but prepregnancy BMI is similar. Short sleep duration was not predictive of epidural use, postpartum haemorrhage, umbilical cord arterial blood pH or base excess or newborn Apgar score.

**Table 3:** Reported Night Sleep Duration and Secondary Outcomes After Labour Induction.

Variable	Short Sleep Duration (n = 102)	Good Sleep Duration (n = 114)	P value	RR (95% CI)
Induction to delivery Interval (hours, mean ± SD)^*^	23.9 ± 13.3	19.9 ± 12.6	0.036	
Prepregnancy BMI (kg/m^2^, mean ± SD)^†^	25.4 ± 5.4	24.8 ± 6.0	0.444	
Gestational Weight Gain (kg, mean ± SD)^†^	14.4 ± 5.9	12.1 ± 5.3	0.003	
Bishop Score (median [IQR])	2 [1–2]	2 [1–3]	0.194	
Birth Weight (kg, mean ± SD)	3.1 ± 0.5	3.0 ± 0.4	0.104	
Low birth weight (<2.5kg vs. ≥ 2.5)	6 (5.9)	16 (14.0)	0.048	0.4 (0.2–1.0)
High birth weight (≥3.6 kg vs. <3.6 kg)^‡^	14 (13.7)	9 (7.9)	0.165	1.7 (0.8 – 3.8)
Epidural Use	19 (18.6)	16 (14.0)	0.360	1.3 (0.7–2.4)
Estimated blood loss (ml, mean ± SD)^§^	339.8 ± 228.7	312.3 ± 200.8	0.353	
>500 mls	17 (16.8)	10 (9.1)	0.093	1.9 (0.9 – 3.9)
**Umbilical cord arterial blood**				
pH (mean ± SD)^||^	7.3 ± 0.1	7.3 ± 0.1	0.824	
Cord pH < 7	1 (1.0)	1 (0.9)	0.926	1.1 (0.1–18.0)
Cord Base deficit (mean ± SD)^¶^	.3.2 ± 3.1	.3.5 ± 2.7	0.480	
Base deficit <8 mmol/L	7 (7.0)	7 (6.2)	0.813	1.1 (0.4–3.1)
**Apgar Score**				
1 min < 4	2 (2.0)	1 (0.9)	0.497	2.2 (0.2–24.3)

Values are stated as n (%), median [Interquartile Range, IQR] or mean ± standard deviation. Analysis was by t-test for continuous. variables, Mann-Whitney U Test for ordinal data, and Chi-square test for categorical data. *n = 191 (25 cases excluded for caesarean section for non reassuring fetal status. ^†^n = 205 (11 women were unable to recall prepregnancy weight and gestational weight gain. ^‡^3.6 kg = 90th centile of birth weight. ^§^n = 211 (5 cases of missing data). ^||^n = 214 (2 cases of missing data). ^¶^n = 213 (3 cases of missing data). ^#^Relative risk cannot be generated as observed count is 0.

## Discussion

We find that Caesarean delivery after labour induction is associated with short sleep duration (<6 hours night sleep reported) after adjustment for parity, pre-pregnancy BMI, bishop score, and birth weight AOR 2.4, 95% CI 1.1-5.0, P = 0.026 (Table 2). Sensitivity analysis incorporating 2 additional variables, ethnicity and Berlin OSA, shows no material change to the predictive value of short sleep duration (AOR 2.3, 95% CI 1.0-5.1, P = 0.039) whilst the finding for OSA shows severe attenuation (AOR 1.2, 95% CI 0.4-3.2, P = 0.782) after adjustment. The induction to delivery interval is longer by 4 hours on average in women with short sleep duration but other adverse maternal and fetal outcomes were similar (Table 3). Our findings suggest the primacy of sleep duration over other sleep measures in the prediction of poor labour induction outcome.

There is limited literature on the impact of sleep on labour induction outcome. We performed a PubMed (http://www.ncbi.nlm.nih.gov/pubmed) search on the 21st August 2016 with the terms “sleep and labour induction or sleep and labour induction” −46 articles were retrieved. We found only one paper that reported a secondary analysis (relative risk not reported) of sleep duration in a subset population who had labour induction^23^.

We derived RR 3.2 for all-case Caesarean delivery in pregnant with women short sleep duration (based on approximate 50^th^ centile sleep duration cut-offs in their population) from a seminal 2004 paper^4^ and applied that in our sample size calculation. Their study population of 131 was exclusively nulliparous with sleep assessed by actigraph whilst ours had 216 women; nulliparous to parous split of 55% to 45% with self-reported night sleep duration. Poor sleep duration women in our study has AOR 2.4 for Caesarean delivery after labour induction. In exploratory analysis, nulliparous women with short sleep duration in our study has AOR 2.8 for Caesarean delivery after labour induction. The findings on the impact size of short sleep duration against Caesarean delivery across our study and theirs are broadly similar. Our longer induction to delivery time in short sleep duration women is also consistent with their finding that severely disrupted sleep is associated with longer labours.

A 2012 report on the effects of sleep quality and duration in late pregnancy on labour and fetal outcome states that in most women with more than 8 hours sleep had normal vaginal delivery with induction^23^. The authors did not report on relative risk and it was not possible on reading the paper to calculate it. 66.4% of their study population reported more than 8 of sleep compared to only 13.9% in our population.

A 2016 report on a longitudinal study of 688 healthy Chinese women with a 55.5% Caesarean delivery rate finds sleep quality particularly in the second trimester to be strongly associated with Caesarean delivery. Short sleep time is not associated with Caesarean delivery but with preterm births^24^. These findings are in contrast to ours that Caesarean delivery after labour induction is predicted by short sleep duration but not poor sleep quality. For their healthy population of nulliparas and multiparas, the Caesarean delivery rate is a relatively high 55.5%^24^ suggesting that their considerations to proceed with a Caesarean is likely to be substantially different from ours.

In a 2014 report of a prospective study with 120 third trimester women using PSQI questionnaire poor sleep quality was significantly associated with vacuum-assisted delivery but not significantly associated with Caesarean section^25^. Our sleep quality using PSQI findings similarly show no association with Caesarean delivery after labour induction.

A 2014 meta-analysis on obstructive sleep apnoea and the risk of perinatal outcomes involving 5 cohort studies with 977 women reports a Caesarean delivery RR 1.87; 95% CI 1.52 to 2.29^8^. In our study, the binomial analysis for obstructive sleep apnoea was a non-significant RR 1.6, 95% CI 1.0-2.7 broadly similar to meta-analysis finding but there is severe attenuation after adjusting for short sleep duration and other potential confounders so that OSA was no longer predictive. Our data shows the primacy of short sleep duration over OSA and other sleep related measures as a predictor of failed induction.

Other studies have not demonstrated a significant association between poor sleep measures and adverse pregnancy outcome; sleep deprivation (<6 hours) was not a significant predictor of Caesarean section after adjustment^26^, and sleep duration was not associated to delivery mode^27^. None of these studies evaluated women undergoing labour induction though most used similar sleep measurement tools.

To our knowledge, the underlying mechanism through which sleep factors can directly impact on adverse pregnancy outcome and specific to our study, on Caesarean delivery and prolongation of labour after labour induction is not known and an interesting area for future research.

For our strength, we evaluated poor sleep duration as a primary predictor alongside a range of sleep measures (sleep quality, obstructive sleep apnoea, daytime sleepiness, restless leg and insomnia) with adjustment as appropriate. The interviews were done by 2 investigators (AT and AD) with a standardised method. Recruitment uptake is high, 94% of those eligible and approached agreed to participate. All participants completed their interview. Our data set is replete. In our multivariable logistic regression model we incorporated the significant variables on binomial analysis. Our study is powered. There were 52 Caesarean deliveries; our primary multivariable regression analysis incorporated 5 predictor variables (P < 0.05), within the 10 events per variable rule for logistic analysis (which may be too conservative^12^). Our sensitivity analysis incorporating all predictor variables P < 0.1 comprised 7 variables in the model.

As for our limitations, we used a subjective measure with night sleep duration self- reported in integer hours and hence potentially open to bias. Questionnaire-derived reports of sleep hours in pregnancy may not reflect objectively measured sleep time^28^; however self-reported sleep hours is commonly used in pregnancy studies^2,3^. Recent population based studies investigating adverse pregnancy outcome including Caesarean delivery to sleep have used less precise retrospective hospital records^29^ and electronic perinatal record^30^ to identify maternal sleep issues. We planned analysis based on 50^th^ centile cut-offs to minimize reliance on absolute sleep duration as we are aware that studies on short sleep duration to adverse pregnancy outcomes have reported different absolute cut-offs of 5 hours or less^7^, less than 6 hours^4^, 6 hours or less^6^, less than 7 hours^5^ and less than 8 hours^23^. Although extensively used in the ‘sleep in pregnancy’ literature, some of the questionnaires we used have not been validated for sleep disorders in pregnancy^21^ or more specifically in late pregnancy^18,22^. PSQI is validated in the last month of pregnancy^15^ and Berlin questionnaire is predictive in later pregnancy^20^. It has also been suggested that studies of sleep duration effects on labour and pregnancy outcomes require a consideration of the amount of both daytime and night time sleep^27^.

## Conclusion

Caesarean delivery after labour induction and a longer induction to delivery interval are associated with reported short night sleep duration in the last month.

## Electronic supplementary material

Supplementary Tables 1 and 2
