# Supplementary material for: The Impact of Self-Reported Sleep on Caesarean Delivery in Women Undergoing Induction of Labour: A Prospective Study
Source: Sci Rep. 2017 Sep 26;7:12339. doi: 10.1038/s41598-017-12410-7 (PMC5615086; doi:10.1038/s41598-017-12410-7)
Supplement: Supplementary file 1 — Supplementary Tables 1 and 2 [file 41598_2017_12410_MOESM1_ESM.doc]

**The Impact of Self-Reported Sleep on Caesarean Delivery in Women Undergoing Induction of Labour: A Prospective Study**

Aimee Chuin Ai Teong^1 + a^, Annabella Xinhui Diong^1 + b^, Siti Zawiah Omar^2 c^, and Peng Chiong Tan^2, d^,

^1^Faculty of Medicine, University of Malaya, Lembah Pantai, Kuala Lumpur, Malaysia

^2^Department of Obstetrics and Gynaecology, Faculty of Medicine, University of Malaya, Lembah Pantai, Kuala Lumpur, Malaysia

^+^Joint first authors

*Corresponding author

^a^[aimee.teong@gmail.com](mailto:aimee.teong@gmail.com)

^b^[annabelladiong@gmail.com](mailto:annabelladiong@gmail.com)

^c^[szawiah@um.edu.my](mailto:szawiah@um.edu.my)

^d^[pctan@um.edu.my](mailto:pctan@um.edu.my)

| **Supplementary Table 1 : Sensitivity Analysis Using All Variables P < 0.1 On Binomial Analysis For The Prediction Of Caesarean Delivery After Labour Induction** | | |
| --- | --- | --- |
| **Variable** | Multivariable Logistic Regression Analysis | |
|  | **AOR (95%CI)** | **P value** |
| **Nulliparity** | 12.1 (4.2 - 35.0) | <0.001 |
| **Ethnicity** |  |  |
| Malay | 2.4 (0.6 - 9.1) | 0.214 |
| Chinese | 1 |  |
| Indian | 3.5 (0.4 - 28.3) | 0.242 |
| Others | 4.1 (1.0 - 17.9) | 0.057 |
| **Prepregnancy BMI** | 1.1 (1.0 - 1.2) | 0.016 |
| **Bishop score** | 1.0 (0.7 - 1.3) | 0.755 |
| **Sleep Duration** |  |  |
| Short Sleep Duration < 6 hours | 2.3 (1.0 - 5.1) | 0.039 |
| Good Sleep Duration ≥ 6 hours |  |  |
| **Berlin Risk of Sleep Apnea** |  |  |
| High Risk | 1.2 (0.4 - 3.2) | 0.782 |
| Low Risk |  |  |
| **Birth Weight (per 100 grams unit)** | 1.1 (1.0 - 1.2) | 0.009 |
|  |  |  |
| Multivariable logistic regression analysis was performed using all 7 predictor variables with a crude p < 0.1. Adjusted odd ratio is shown for all variables used in the model. | | |

| **Supplementary Table 2 : Nulliparous Women's Characteristics For the Prediction of Caesarean Delivery after Labour Induction** | | | | | | | | |
| --- | --- | --- | --- | --- | --- | --- | --- | --- |
| Variable | Cesarean Delivery (n=43) | Vaginal Delivery (n=75) | P Value | RR (95% CI) | Multivariable Logistic Regression Analysis 1 | | Multivariable Logistic Regression Analysis 2 | |
|  |  |  |  |  | AOR (95%CI) | P value | AOR (95%CI) | P value |
| **Age (years, mean ± SD)** | 30.2 ± 3.7 | 29.4 ± 4.0 | 0.285 |  |  |  |  |  |
| **Ethnicity** |  |  |  |  |  |  |  |  |
| Malay | 22 (51.2) | 43 (57.3) | 0.040 |  |  |  | 2.3 (0.5 - 10.4) | 0.282 |
| Chinese | 4 (9.3) | 15 (20.0) |  |  |  |  | 1 |  |
| Indian | 16 (37.2) | 12 (16.0) |  |  |  |  | 2.4 (0.2 - 36.0) | 0.528 |
| Others | 1 (2.3) | 5 (6.7) |  |  |  |  | 5.2 (1.0 - 26.7) | 0.048 |
| **Prepregnancy BMI (kg/m^2^, mean ± SD)^*^** | 26.8 ± 6.1 | 23.4 ± 4.4 | 0.003 |  | 1.1 (1.1 - 1.3) | 0.002 | 1.1 (1.0 - 1.3) | 0.017 |
| **Gestational Weight Gain (kg, mean ± SD)^*^** | 13.3 ± 5.6 | 14.5 ± 5.1 | 0.254 |  |  |  |  |  |
| **Gestational age (weeks, mean ± SD)** | 39.5 ± 1.5 | 39.2 ± 1.3 | 0.349 |  |  |  |  |  |
| **Indications for Induction of Labor** |  |  |  |  |  |  |  |  |
| Diabetes mellitus | 20 (46.5) | 26 (34.7) | 0.050 |  |  |  |  |  |
| Prolonged pregnancy | 14 (32.6) | 16 (21.3) |  |  |  |  |  |  |
| Hypertension | 6 (14.0) | 10 (13.3) |  |  |  |  |  |  |
| Non-reassuring fetal status ^†^ | 3 (7.0) | 20 (26.7) |  |  |  |  |  |  |
| Others | 0 (0.0) | 3 (4.0) |  |  |  |  |  |  |
| **Bishop score (median [IQR])** | 1 [1-2] | 2 [1-3] | 0.032 |  | 0.9 ( 0.6 - 1.3) | 0.583 | 0.9 (0.6 - 1.3) | 0.491 |
| **Mode of labor induction** |  |  |  |  |  |  |  |  |
| Vaginal dinoprostone^‡^ | 42 (97.7) | 72 (96.0) | 0.629 | 1.5 (0.3 - 8.2) |  |  |  |  |
| Amniotomy | 1 (2.3) | 3 (4.0) |  |  |  |  |  |  |
| **Reported Night Sleep** |  |  |  |  |  |  |  |  |
| Duration (hours, median [IQR]) | 5 [4 - 6] | 6 [5 - 7] | 0.018 |  |  |  |  |  |
| Short Sleep Duration < 6 hours | 27 (62.8) | 28 (37.3) | 0.008 | 1.9 (1.2 - 3.2) | 2.9 (1.2 - 6.9) | 0.019 | 2.8 (1.1 - 7.1) | 0.031 |
| Good Sleep Duration ≥ 6 hours | 16 (37.2) | 47 (62.7) |  |  |  |  |  |  |
| < 4 hours | 13 (30.2) | 12 (16.0) | 0.010^§^ |  |  |  |  |  |
| 5 hours | 14 (32.6) | 16 (21.3) |  |  |  |  |  |  |
| 6 hours | 8 (18.6) | 22 (29.3) |  |  |  |  |  |  |
| ≥ 7 hours | 8 (18.6) | 25 (33.3) |  |  |  |  |  |  |
| **Pittsburgh Sleep Quality Index** |  |  |  |  |  |  |  |  |
| Poor Sleep Quality | 30 (69.8) | 51 (68.0) | 0.842 | 1.1 (0.7 - 1.8) |  |  |  |  |
| Good Sleep Quality | 13 (30.2) | 24 (32.0) |  |  |  |  |  |  |
| **Berlin Risk of Sleep Apnea^ǁ^** |  |  |  |  |  |  |  |  |
| High Risk | 12 (29.3) | 10 (13.3) | 0.036 | 1.8 (1.1 - 2.9) |  |  | 1.2 (0.4 - 4.3) | 0.745 |
| Low Risk | 29 (70.7) | 65 (86.7) |  |  |  |  |  |  |
| **Epworth Sleepiness Scale** |  |  |  |  |  |  |  |  |
| Daytime Sleepiness | 8 (18.6) | 11 (14.7) | 0.575 | 1.2 (0.7 - 2.2) |  |  |  |  |
| Normal | 35 (81.4) | 64 (85.3) |  |  |  |  |  |  |
| **Restless Leg Syndrome** |  |  |  |  |  |  |  |  |
| Present | 23 (53.5) | 42 (56.0) | 0.792 | 0.9 (0.6 - 1.5) |  |  |  |  |
| Absent | 20 (46.5) | 33 (44.0) |  |  |  |  |  |  |
| **Insomnia Symptom Questionnaire** |  |  |  |  |  |  |  |  |
| Insomnia Disorder | 10 (23.3) | 17 (22.7) | 0.942 | 1.0 (0.6 - 1.8) |  |  |  |  |
| None | 33 (76.7) | 58 (77.3) |  |  |  |  |  |  |
| **Birth Weight (100 grams, mean ± SD)** | 31.0 ± 4.2 | 29.0 ± 4.1 | 0.012 |  | 1.2 (1.0 - 1.3) | 0.012 | 1.2 (1.0 - 1.3) | 0.012 |
| Values are stated as n (%), median [Interquartile Range, IQR] or mean ± standard deviation. Analysis was by t-test for continuous variables, Mann-Whitney U Test for ordinal data, Chi-square test for categorical data, and chi-square test for trend for sequential categorical data. Multivariable logistic regression analysis 1 incorporated 4 predictor variables - prepregnant BMI, Bishop Score, dichotomized sleep duration and birth weight. Multivariable logistic regression analysis 2 incorporated 6 predictor variables - prepregnant BMI, Bishop Score, dichotomized sleep duration, birth weight, obstructive sleep apnoea and ethnicity. Adjusted odd ratio is shown for all variables used in the models. | | | | | | | | |
| ^1^ n = 114 (4 women were unable to recall prepregnancy weight / gestational weight gain / height) | | | | | | | | |
| ^2^ Includes oligohydramnios, suspected IUGR, reduced fetal movement and suboptimal umbilical artery on Doppler examination. | | | | | | | | |
| ^3^ Includes a single case of ripening with the Foley catheter | | | | | | | | |
| ^4^ p value obtained from Chi Square for trend | | | | | | | | |
| ^5^ n = 116 (Unable to classify 2 women into high or low risk for sleep apnea due to missing BMI data) | | | | | | | | |
